# Osteoporosis-related fractures in men and women with established and early rheumatoid arthritis: predictors and risk compared with the general population

**DOI:** 10.1186/s41927-023-00354-7

**Published:** 2023-09-08

**Authors:** Lisa Theander, Lennart T.H. Jacobsson, Carl Turesson

**Affiliations:** 1 Rheumatology, Department of Clinical Sciences, Malmö, Lund University, Jan Waldenströms gata 1B, 205 02 Malmö, Sweden; 2 Department of Rheumatology and Inflammation Research, Sahlgrenska Academy at Gothenburg University, Gothenburg, Sweden; 3 Department of Rheumatology, Skåne University Hospital, Malmö and Lund, Malmö, Sweden

**Keywords:** Rheumatoid arthritis, Fractures, Osteoporosis

## Abstract

**Objectives:**

To study the risk of osteoporosis-related fractures in a community-based sample of men and women with rheumatoid arthritis (RA) overall, as well as early (< 1 year of disease duration, follow-up time maximum 10 years) and established (RA diagnosis since ≥ 5 years on July 1, 1997) RA, compared with the general population. To study potential risk factors for fractures in patients with RA from baseline questionnaire data.

**Methods:**

A community-based cohort of patients with RA (n = 1928) was studied and compared to matched general population controls. Information on osteoporosis-related fractures (hip, proximal upper arm, distal forearm and vertebral fractures) during the period July 1, 1997 to December 31, 2017 was obtained by linkage to the Swedish National Inpatient Register and the Cause of Death Register. The incidence of fractures was estimated in patients and controls. Cox regression models were used to assess the relation between RA and the risk of fractures and to assess potential predictors of fractures in RA patients. Analyses were stratified by sex, and performed in all patients with RA, and in subsets with early and established RA.

**Results:**

The overall incidence of osteoporosis-related fractures in the RA cohort was 10.6 per 1000 person-years (95% CI 9.31; 12.0). There was an increased risk of fractures overall in both men (hazard ratio (HR) 1.55, 95% CI 1.03; 2.34) and women (HR 1.52; 95% CI 1.27; 1.83) with RA compared to controls, with significantly increased risk also in the hip. No increased risk of osteoporosis-related fractures overall was seen in patients with early RA (HR 1.01, 95% CI 0.69; 1.49). Higher age, longer duration of RA, higher HAQ scores and higher scores in the visual analogue scale for global health were predictors of fractures.

**Conclusion:**

Both men and women with RA were at increased risk of osteoporosis-related fractures. Patients with early RA did not have significantly increased risk during the first 10 years of disease in this study.

**Supplementary Information:**

The online version contains supplementary material available at 10.1186/s41927-023-00354-7.

## Introduction

Rheumatoid arthritis (RA) is a systemic autoimmune disease associated with many comorbidities including osteoporosis and fragility fractures. Clinical evident osteoporosis (bone mineral density T-score<-2.5) has been observed in a substantial proportion of RA patients and has been viewed partly as a result of chronic inflammation, treatment with glucocorticosteroids, D-vitamin deficiency and immobility [1, 2]. In addition to risk factors of osteoporosis, patients with RA also have an increased risk of falling [3, 4], which predisposes them for fractures. Earlier studies and meta-analyses have shown that RA patients have about doubled risk for fractures compared to control populations [2, 5–7]. In studies comparing the incidence of fractures over time, no obvious reduction has been seen during recent years [5, 8, 9] despite better availability of treatment for both RA and osteoporosis. Indeed the “treatment gap” – i.e. the proportion of individuals with high risk of fracture with adequate treatment vs. the proportion of individuals with high risk of fracture without adequate treatment, for RA patients is getting increasing attention in literature [1, 2, 7, 10]. RA-related factors that have been associated with increased fracture risk are high disease activity, long disease duration and disability measured by the Health Assessment Questionnaire (HAQ) scores [2, 5, 6]. These are factors that reflect the effects of both chronic inflammation and level of physical activity on bone health. Established predictors of fractures in the general population, such as high age, low BMI, postmenopausal status in women, and prior fractures, also apply to patients with RA [5]. Loss of bone mass has been seen soon after RA diagnosis [11–13], but less is known about the risk of fractures in the early years after RA diagnosis. Nyhäll-Wåhlin et al. found an increased risk of fractures in patients followed from the time of diagnosis to a maximum of 8 years later [8]. Van Staa et al. found a significantly increased risk already during the first 2 years, although the risk increased with disease duration [14]. As data on risk of fracture in early RA is limited, this needs further investigation.

Men and women are affected by osteoporosis in different ways. Women typically experience bone loss earlier in life and at a faster rate than men due to hormonal changes at the time of menopause. Osteoporosis in men on the other hand is often overlooked and undertreated [15]. By examining the risks of fractures separately in men and women it is possible to find and highlight potential differences in risk and risk factors between men and women.

The aims of this study were (1) to examine the incidence of osteoporosis-related fractures (hip, proximal upper arm, distal forearm and vertebral fractures) in men and women with RA and compare it to that of the general population, with subanalyses of patients with a short disease duration, and (2) to investigate potential baseline predictors of such fractures in patients with RA. As hip fractures lead to a greater morbidity burden and are more reliably captured in the inpatient register than other osteoporosis-related fractures, we also investigated incidence and predictors of hip fractures separately.

## Patients and methods

### Patients and controls

A community-based cohort of patients with RA (n = 1928) was investigated. The cohort was based on a register of all known patients with RA in Malmö, Sweden, established in 1997. The patients were recruited from the rheumatology outpatient clinic of Malmö University Hospital and from all rheumatologists in private practice in Malmö. At the time of establishment, the register covered about 95% of all patients with RA in the area [16]. The register was extended with newly diagnosed patients until the year of 2006, as previously described [17, 18]. All patients were seen by a rheumatologist and diagnosed after fulfilment of the 1987 American College of Rheumatology criteria for RA.

Four controls without RA diagnosis per patient, individually matched for age at inclusion of the case in the register (+/- 1 year), sex and residential area were identified using the national census register from the general population. Retrieval of matched controls was performed by Statistics Sweden.

### Clinical characteristics of patients

In 1997, 2002, 2005, and 2009, the patients received questionnaires including the Health Assessment Questionnaire (HAQ), visual analogue scales (VAS) for current pain and global health, and questions on current and previous antirheumatic treatment. At least one completed questionnaire was obtained from 1523 (79%) of the included patients after one reminder during the study period. Information on treatment with biologic Disease-modifying Antirheumatic Drugs (bDMARDs) from study start to December 31, 2016 was obtained from the South Swedish Arthritis Treatment Group (SSATG) regional register on bDMARD treatment [19] and the Swedish Rheumatology Quality register [20], which includes national data on bDMARDs. Data on Rheumatoid Factor (RF) tests were retrieved from the databases of the two clinical immunology laboratories in the area. Patients with ≥ 1 positive RF test at any time were considered positive.

### Identification of fractures

Information on fractures in patients and controls during the period July 1, 1997 to December 31, 2017 was obtained by linkage to the Swedish National Inpatient Register and the Cause of Death Register. Fractures of the hip, proximal upper arm, distal forearm and vertebra were identified based on ICD-9 and ICD-10 diagnostic codes from in-patient care (Supplementary Tables 1, Additional file 1). High-energy traumatic fractures during the study period were identified using ICD-10 external cause codes [21].

### Statistics

#### Comparison of fracture rates in patients with RA and controls – main analyses

Patients and controls with identified fractures before study start (1. July 1997 or date of RA diagnosis/corresponding index date for controls) were excluded from the analyses described below. Only the first incident fracture of the corresponding analysis was used in this study. In all analyses patients and controls were censored for death, emigration or end of study, 31 December 2017. The incidence of osteoporosis-related fractures in total (hip, proximal upper arm, distal forearm and vertebral fractures) and hip fractures specifically was estimated in RA patients and controls, overall and stratified by sex. The 95% confidence intervals (CI) for incidence rates and incidence rate ratios (IRRs) were estimated using the Poisson distribution ratio. For subsets with < 5 events in RA patients, IRRs were not estimated.

The relation between RA and the risk of osteoporosis-related fractures in total and hip fractures was also assessed using Cox regression models and presented as hazard ratios. In order to take the matched design into account, the group variable identifying each case and its matched controls was entered as a stratum variable. Separate analyses for the 10 first years of disease in patients that were included in the register with < 1 year of disease duration in 1997–2006 were performed to examine fracture incidence and hazard ratios in early RA. For comparison, the same analyses were performed in patients with RA diagnosis for ≥ 5 years on July 1, 1997. Early and established disease was also studied in a multivariable model where the interaction between RA and the status of disease (early vs. established) was analysed. For subsets with < 10 events in RA patients no Cox regression analyses were performed. Although the expected numbers of fractures were lower, these analyses were also performed for the subsets of vertebral, proximal upper arm and distal forearm fractures.

#### Sensitivity analyses

We performed Cox regression analyses where fractures with external cause ICD-10 codes for high-energy trauma during the study period were excluded [21]. In further sensitivity analyses for the risk of osteoporosis-related fractures overall in RA patients compared to matched controls, patients with osteoporosis-related fractures occurring before study start were not excluded. Instead, such fractures were adjusted for as a covariate in cox regression models.

#### Prediction of fractures in patients with RA

The impact of baseline characteristics (age, duration of RA, RF status, HAQ score, VAS for current pain and patient’s assessment of global health and treatment with Methotrexate, bDMARDs and Prednisolone at the date of the first answered questionnaire) on the risk of fractures in RA patients was examined in bivariate and age-adjusted Cox regression models. In these analyses the date of the first answered questionnaire was used as start of follow-up and the time to first fracture was investigated. Patients with fractures before start of follow-up were excluded. For all models proportional hazards assumptions were evaluated using log-minus-log plots and Schoenfeld residuals. The analyses were performed using SPSS version 25.

#### Ethics

This study was approved by the Regional Ethical Review Board for southern Sweden (Lund, Sweden, LU 336-01, LU 2016/923). Informed consent was waived by the Regional Ethical Review Board for southern Sweden and was not obtained for the present study. The study was conducted according to the principles of the Helsinki Declaration.

## Results

### Patients and baseline characteristics

Of the 1928 patients included in the study, 1401 (73%) were women. Mean age at inclusion was 60.5 (standard deviation (SD) 14.7) years in men and 59.5 (SD 15.9) years in women (age range 19–89 years and 16–93 years respectively). Median duration of disease was 3 (interquartile range 0–14) years in both men and women at inclusion. A total of 13 (2.5%) of the included men and 81 (5.8%) of the women with RA had had at least one of the studied fractures before study start. The corresponding numbers for controls was 33 (1.6%) in men and 186 (3.3%) in women. A total of 1022 (53.0%) of the included patients and 3649 (47.7%) of the controls were censored for death or emigration before December 31, 2017 (end of study). Of the men with RA, 105 (26%) and of the women, 273 (25%) reported treatment with glucocorticosteroids at the time of their first answered questionnaire. A total of 585 RA patients (30.3%) were treated with biologic DMARDs at any time during the study period, up to December 31, 2016. Further demographic and clinical baseline characteristics of the study cohort are shown in Table 1. Patients with newly diagnosed RA (RA duration < 1 year at baseline, n = 738) were on average younger and less frequently RF positive, had lower HAQ scores and were more often treated with methotrexate and biologic DMARDs at the time of their first answered questionnaire (Table 1). In the group of patients with fractures before study start both men and women were older, had longer duration of disease, a somewhat higher proportion with positive RF and scored higher in disease activity measures than patients without previous fractures (Supplementary Tables 2, Additional file 1). Patients who had not returned any completed questionnaires during the study period were older and had had more fractures before study start (Supplementary Tables 3, Additional file 1).

**Table 1:** Baseline characteristics for patients in the total cohort, with early and established RA

Total cohort	Men	Women	All
Number	527	1401	1928
Age (years) mean (SD)	60.5 (14.7)	59.5 (15.9)	59.8 (15.6)
RA duration at inclusion (years), median (IQR)	3 (0–14)	3 (0–14)	3 (0–14)
RA duration at first questionnaire (years), median (IQR)	7 (4–17)	7 (4–17)	7 (4–17)
RF-positive n (%)	322 (73.5)	855 (73.3)	1177 (73.3)
HAQ mean (SD)*	0.75 (0.68)	1.1 (0.76)	0.97 (0.75)
VAS pain (mm) mean (SD)*	37.9 (27.4)	44.5 (26.5)	42.7 (26.9)
VAS global health (mm) mean (SD)*	38.2 (26.4)	42.7 (26.2)	41.5 (26.3)
Methotrexate n (%)*	189 (46.1)	479 (43.0)	668 (43.9)
csDMARDs other than Methotrexate n (%)*	114 (27.8)	321 (28.8)	435 (28.6)
bDMARDs n (%)*	42 (10.2)	121 (10.9)	163 (10.7)
Prednisolone n (%)*	105 (25.6)	273 (24.5)	378 (24.8)
Previous osteoporosis-related fracture n (%)	13 (2.5)	81 (5.8)	94 (4.9)
**Early RA** ^**¶**^	**Men**	**Women**	**All**
Number	211	527	738
Age (years) mean (SD)	57.9 (14.7)	55.4 (16.8)	56.1 (16.3)
RA duration at inclusion (years), median (IQR)	< 1 (< 1-<1)	< 1 (< 1-<1)	< 1 (< 1-<1)
RA duration at first questionnaire (years), median (IQR)	4 (2–5)	4 (2–5)	4 (2–5)
RF-positive n (%)	127 (67.7)	303 (66.3)	430 (66.7)
HAQ mean (SD)*	0.63 (0.59)	0.77 (0.57)	0.73 (0.58)
VAS pain (mm) mean (SD)*	37.3 (27.1)	40.1 (24.9)	39.4 (25.5)
VAS global health (mm) mean (SD)*	38.1 (26.6)	39.1 (24.4)	38.9 (25.0)
Methotrexate n (%)*	101 (67.3)	256 (62.6)	357 (63.9)
csDMARDs other than Methotrexate n (%)*	41 (27.3)	105 (25.7)	146 (26.1)
bDMARDs n (%)*	26 (17.3)	72 (17.6)	98 (17.5)
Prednisolone n (%)*	37 (24.7)	94 (23.0)	131 (23.4)
Previous osteoporosis-related fracture n (%)	1 (0.5)	12 (2.3)	13 (1.8)
**Established RA** ^**¶¶**^	**Men**	**Women**	**All**
Number	237	638	875
Age (years) mean (SD)	64.1 (13.3)	63.4 (13.9)	63.6 (13.7)
RA duration at inclusion (years), median (IQR)	14 (9–22)	15 (9–26)	15 (9–25)
RA duration at first questionnaire (years), median (IQR)	18 (11–25)	18 (11–27)	18 (11–27)
RF-positive n (%)	141 (79.2)	402 (80.9)	543 (80.4)
HAQ mean (SD)*	0.96 (0.74)	1.35 (0.80)	1.24 (0.80)
VAS pain (mm) mean (SD)*	41.1 (27.7)	48.7 (27.2)	46.6 (27.6)
VAS global health (mm) mean (SD)*	41.3 (27.0)	46.5 (27.2)	45.0 (27.2)
Methotrexate n (%)*	62 (32.3)	157 (30.8)	219 (31.2)
csDMARDs other than Methotrexate n (%)*	52 (27.1)	149 (29.3)	201 (28.7)
bDMARDs n (%)*	13 (6.8)	38 (7.5)	51 (7.3)
Prednisolone n (%)*	56 (29.2)	130 (25.5)	186 (26.5)
Previous osteoporosis-related fracture n (%)	10 (4.2)	61 (9.6)	71 (8.1)

*At the date of the first available questionnaire

In the total cohort at least one questionnaire was answered by 1523 patients. Of these 10 were missing HAQ, 50 missing VAS pain and 52 missing VAS global health

SD: Standard Deviation; IQR: Interquartile Range; RA: Rheumatoid Arthritis; RF: Rheumatoid Factor; HAQ: Health Assessment Questionnaire; VAS: Visual Analogue Scale; csDMARDs: conventional synthetic Disease-modifying Antirheumatic Drugs; bDMARDs: biologic Disease-modifying Antirheumatic Drugs

¶Early RA: newly diagnosed (within 1 year) patients in 1997 or later, with follow-up time maximal 10 years

¶¶Established RA: patients with RA diagnosis for ≥ 5 years at study start (1997)

### Incidence and risk of fractures in RA patients without previous fractures compared with controls

A total of 51 (10.2%) men and 202 (15.8%) women with RA suffered from at least one of the studied fractures during the study period, compared with 118 (6.6%) male and 602 (12.3%) female controls (Table 2). The mean period of follow-up was 12.7 years in men and 13.6 years in women with RA, and 12.2 and 13.8 years in male and female controls respectively. The corresponding incidence rates per 1000 person-years at risk (PYR) were 7.86 in men with RA compared to 4.70 in male controls and 11.6 in women with RA compared to 8.27 in female controls (Table 2). The overall incidence of fractures in the RA cohort was 10.6 per 1000 PYR (95% CI 9.31; 12.0).

**Table 2:** Number and incidence of osteoporosis-related fractures in RA patients and matched controls

		Men		Women		All	
Fracture	Total cohort	RA patients	Controls	RA patients	Controls	RA patients	Controls
Hip	n (%)	43 (8.5)	92 (5.1)	147 (11.2)	463 (9.3)	190 (10.5)	555 (8.2)
	Incidence/1000 PY (95% CI)	6.56 (4.75; 8.84)	3.63 (2.93; 4.45)	8.16 (6.89; 9.59)	6.21 (5.66; 6.81)	7.73 (6.67; 8.91)	5.56 (5.11; 6.04)
Fractures in total	n (%)	51 (10.2)	118 (6.6)	202 (15.8)	602 (12.3)	253 (14.2)	720 (10.8)
	Incidence/1000 PY (95% CI)	7.86 (5.85; 10.33)	4.70 (3.89; 5.62)	11.58 (10.04; 13.29)	8.27 (7.62; 8.95)	10.57 (9.31; 11.96)	7.35 (6.82; 7.91)
**Fracture**	Early RA*	**RA patients**	**Controls**	**RA patients**	**Controls**	**RA patients**	**Controls**
Hip	n (%)	6 (3.0)	24 (3.0)	16 (3.2)	68 (3.4)	22 (3.1)	92 (3.3)
	Incidence/1000 PY (95% CI)	3.41 (1.25; 7.43)	3.51 (2.25; 5.22)	3.48 (1.22; 5.64)	3.73 (2.90; 4.73)	3.46 (2.17; 5.24)	3.67 (2.96; 4.50)
Fractures in total	n (%)	7 (3.4)	33 (4.2)	29 (5.8)	101 (5.1)	36 (5.1)	134 (4.8)
	Incidence/1000 PY (95% CI)	4.00 (1.61; 8.25)	4.89 (3.36; 6.86)	6.40 (4.29; 9.19)	5.64 (4.60; 6.86)	5.73 (4.02; 7.94)	5.44 (4.55; 6.44)
**Fracture**	Established RA**	**RA patients**	**Controls**	**RA patients**	**Controls**	**RA patients**	**Controls**
Hip	n (%)	31 (14.0)	39 (5.4)	82 (14.0)	257 (12.2)	113 (14.0)	296 (10.5)
	Incidence/1000 PY (95% CI)	11.35 (7.71; 16.11)	3.65 (2.60; 4.99)	10.79 (8.58; 13.40)	7.84 (6.91; 8.86)	10.94 (9.02; 13.15)	6.81 (6.06; 7.63)
Fractures in total	n (%)	34 (15.5)	46 (6.4)	106 (18.9)	324 (15.6)	140 (17.9)	370 (13.3)
	Incidence/1000 PY (95% CI)	12.55 (8.69; 17.53)	4.32 (3.16; 5.76)	14.64 (11.99; 17.71)	10.15 (9.07; 11.32)	14.07 (11.84; 16.60)	8.69 (7.83; 9.62)

RA: Rheumatoid Arthritis; n: Number; PY: Person Years; CI: Confidence Interval

*Early RA: newly diagnosed (within 1 year) patients in 1997 or later with follow-up time maximal 10 years

**Established RA: patients with RA diagnosis for ≥ 5 years at study start (1997)

Both men and women with RA had increased risk of fractures overall (hazard ratio (HR) 1.55, 95% CI 1.03; 2.34 and HR 1.52, 95% CI 1.27; 1.83, respectively) and of fractures in the hip (HR 1.68, 95% CI 1.05; 2.68 and HR 1.41, 95% CI 1.14; 1.75, respectively) (Table 3). In analyses only including newly diagnosed patients from the year of 1997 or later, with a maximum follow-up time of 10 years, no increased risk of fractures overall or in the hip was seen compared to the matched control population (Table 3). The number of fractures in men with early RA (in total 7 fractures) was too low to be sufficient for Cox regression analyses. Results of patients with more established disease were similar to the results of the total cohort, with exception for the association between RA and hip fractures in men, which was stronger in patients with RA duration of ≥ 5 years at study start (HR 3.77, 95% CI 1.79; 7.96) (Table 3).

**Table 3:** IRR and HR for osteoporosis-related fractures in RA patients compared with matched controls

	INCIDENCE RATE RATIO (95% CI)			HAZARD RATIO (95% CI)		
**Total cohort**	**Men**	**Women**	**All**	**Men**	**Women**	**All**
Hip fracture	**1.81** **(1.23; 2.61)**	**1.31** **(1.08; 1.58)**	**1.39** **(1.17; 1.64)**	**1.68** **(1.05; 2.68)**	**1.41** **(1.14; 1.75)**	**1.46** **(1.20; 1.77)**
Fractures in total	**1.67** **(1.18; 2.33)**	**1.40** **(1.19; 1.64)**	**1.44** **(1.24; 1.66)**	**1.55** **(1.03; 2.34)**	**1.52** **(1.27; 1.83)**	**1.53** **(1.29; 1.81)**
Early RA*						
Hip fracture	0.97 (0.33; 2.46)	0.93 (0.50; 1.61)	0.94 (0.56; 1.50)	NA	0.85 (0.49; 1.49)	0.81 (0.50; 1.33)
Fractures in total	0.82 (0.31; 1.88)	1.13 (0.72; 1.72)	1.05 (0.71; 1.53)	NA	1.14 (0.74; 1.75)	1.01 (0.69; 1.49)
Established RA**						
Hip fracture	**3.11** **(1.88; 5.06)**	**1.38** **(1.06; 1.77)**	**1.61** **(1.28; 2.00)**	**3.77** **(1.79; 7.96)**	**1.76** **(1.31; 2.38)**	**1.97** **(1.50; 2.59)**
Fractures in total	**2.90** **(1.81; 4.58)**	**1.44** **(1.15; 1.80)**	**1.62** **(1.32; 1.97)**	**2.99** **(1.57; 5.70)**	**1.77** **(1.36; 2.30)**	**1.91** **(1.50; 2.43)**

Bold text indicates statistically significant results. IRR: Incidence Rate Ratio; HR: Hazard Ration; RA: Rheumatoid Arthritis; CI: Confidence Interval; NA: Not applicable due to < 10 events in patients with RA

*Early RA: newly diagnosed (within 1 year) patients from the year of 1997 with follow-up time maximal 10 years

**Established RA: patients with RA diagnosis for ≥ 5 years at study start (1997)

In supplementary multivariable cox regression models analysing the risk of fractures, the interaction between RA and the status of early or established disease did not reach statistical significance for fractures in total (p = 0.13) or hip fractures specifically (p = 0.07).

Considering other subtypes of fractures (vertebral, proximal upper arm and distal forearm fractures), numbers were lower (Supplementary Tables 4, Additional file 1), but there was a higher rate of proximal upper arm fractures in patients with RA, with similar patterns in the early and established RA subsets. (Supplementary Tables 5, Additional file 1).

In men with RA there were 5 fractures with external cause ICD-10 codes for high-energy trauma during the study period and in women with RA there were 7 (compared to 8 and 27 traumatic fractures in male and female controls respectively). In analyses excluding fractures caused by high-energy trauma, hazard ratios were generally somewhat lower. Although the results in men in the total cohort were no longer significant, the overall trends were similar (Supplementary Tables 6, Additional file 1).

In sensitivity analyses for the risk of osteoporosis-related fractures overall in RA patients compared to matched controls, not excluding patients with osteoporosis-related fractures before study start, but adjusted for such fractures in cox regression models, the results were similar to the main analyses (HR 1.62 (95% CI 1.08; 2.42) in men, HR 1.44 (95% CI 1.21; 1.72) in women and HR 1.47 (95% CI 1.25; 1.72) in the total cohort). In this analysis, previous fracture was significantly associated with further fractures overall after study start in the total cohort (HR 2.10, 95% CI 1.48; 2.98) and in women (HR 2.08, 95% CI 1.45; 2.98). In men there was a similar trend but no statistically significant association (HR 2.64, 95% CI 0.61; 11.45).

### Predictors of fractures in RA patients

Proportional hazards assumptions were fulfilled for most models but not for some of the predictor analyses (Tables 4 and 5 and supplementary Tables 7 and 8, Additional file 1). In unadjusted analyses higher age, longer duration of RA disease, higher HAQ scores and higher scores in the VAS for global health were significantly associated with fractures overall (Table 4). The associations with HAQ scores and VAS for global health reached statistical significance in age-adjusted analyses for all patients, but not when stratified by sex. The results were similar in analyses of hip fractures only (Table 5). There were no consistent associations between treatment with methotrexate, bDMARDs or glucocorticosteroids and fracture risk (Tables 4 and 5).

**Table 4:** Baseline predictors of osteoporosis-related fractures overall in the full RA cohort*

Fractures overall	Men (n 391)		Women (n 1008)		All (n 1399)	
	HR (95% CI)	Age-adjusted HR (95% CI)	HR (95% CI)	Age-adjusted HR (95% CI)	HR (95% CI)	Age-adjusted HR (95% CI)
Age, per 10 years	**3.59** **(2.52; 5.12)**	NA	**2.32 ** **(1.99; 2.71)**	NA	**2.51 ** **(2.18; 2.90)**	NA
RA duration, per 10 years	**1.49 ** **(1.19; 1.87)**	1.23 (0.99; 1.51)	**1.23** **(1.09; 1.39)**	1.08 (0.96; 1.21)	**1.28 ** **(1.15; 1.43)**	1.11 (1.00; 1.22)
RF-positive	1.02 (0.46; 2.23)	1.76 (0.79; 3.90)	NA^1^	NA^1^	1.07 (0.74; 1.53)	1.34 (0.93; 1.93)
HAQ, per SD	**1.68 ** **(1.20; 2.34)**	1.40 (0.99; 1.96)	**1.36 ** **(1.14; 1.63)**	1.11 (0.93; 1.33)	**1.45 ** **(1.24; 1.69)**	**1.20 ** **(1.03; 1.40)**
VAS pain, per SD	1.19 (0.86; 1.65)	1.31 (0.96; 1.80)	1.07 (0.90; 1.27)	1.01 (0.85; 1.20)	1.11 (0.96; 1.29)	1.10 (0.94; 1.28)
VAS global health, per SD	1.36 (0.98; 1.89)	**1.55 ** **(1.11; 2.15)**	**1.20 ** **(1.01; 1.43)**	1.10 (0.93; 1.32)	**1.25 ** **(1.08; 1.46)**	**1.20** **(1.03; 1.40)**
Methotrexate	NA^1^	NA^1^	0.84 (0.60; 1.17)	0.93 (0.66; 1.30)	NA^1^	NA^1^
bDMARDs	0.24 (0.03; 1.77)	0.65 (0.09; 4.85)	0.47 (0.22; 1.01)	1.01 (0.46; 2.19)	**0.41** **(0.20; 0.85)**	0.90 (0.44; 1.85)
Prednisolone	1.68 (0.84; 3.34)	1.78 (0.88; 3.61)	**1.56 ** **(1.09; 2.23)**	1.24 (0.86; 1.77)	**1.58 ** **(1.16; 2.17)**	1.31 (0.95; 1.80)

* Unadjusted and age-adjusted Cox regression analyses

At the date of the first available questionnaire. Bold text indicates statistically significant results

n: number of patients with at least one answered questionnaire after exclusion of patients with fractures before baseline; HR: Hazard Ratio; CI: Confidence Interval; RA: Rheumatoid Arthritis; RF: Rheumatoid Factor; HAQ: Health Assessment Questionnaire; VAS: Visual Analogue Scale; bDMARDs: biologic Disease-modifying Antirheumatic Drugs; SD: Standard Deviation; NA: Not Applicable; NA^1^: Not Applicable since proportional hazards assumptions were not fulfilled

**Table 5:** Baseline predictors of hip fractures in the full RA cohort*

Hip fractures	Men (n 394)		Women (n 1036)		All (n 1430)	
	HR (95% CI)	Age-adjusted HR (95% CI)	HR (95% CI)	Age-adjusted HR (95% CI)	HR (95% CI)	Age-adjusted HR (95% CI)
Age, per 10 years	**5.02 ** **(3.23; 7.82)**	NA	**2.51 ** **(2.09; 3.03)**	NA	**2.85** **(2.39; 3.40)**	NA
RA duration, per 10 years	**1.64 ** **(1.29; 2.08)**	1.23 (0.99; 1.54)	NA^1^	NA^1^	**1.27 ** **(1.12; 1.43)**	1.08 (0.96; 1.21)
RF-positive	1.33 (0.51; 3.50)	2.59 (0.98; 6.88)	NA^1^	NA^1^	1.18 (0.77; 1.82)	**1.55** **(1.01; 2.39)**
HAQ, per SD	**1.72 ** **(1.18; 2.51)**	1.38 (0.93; 2.06)	**1.29 ** **(1.05; 1.59)**	1.03 (0.84; 1.27)	**1.40 ** **(1.17; 1.67)**	1.13 (0.94; 1.34)
VAS pain, per SD	1.14 (0.79; 1.65)	1.26 (0.87; 1.81)	1.10 (0.90; 1.34)	1.03 (0.84; 1.25)	1.12 (0.94; 1.34)	1.09 (0.92; 1.30)
VAS global health, per SD	**1.46** **(1.01; 2.12)**	**1.63** **(1.13; 2.36)**	1.20 (0.99; 1.47)	1.09 (0.89; 1.33)	**1.27** **(1.07; 1.52)**	**1.20 ** **(1.00; 1.43)**
Methotrexate	**0.26** **(0.10; 0.69)**	0.49 (0.18; 1.33)	0.70 (0.47; 1.04)	0.78 (0.52; 1.16)	NA^1^	NA^1^
bDMARDs	0.33 (0.05; 2.45)	1.23 (0.16; 9.49)	**0.18** **(0.04; 0.73)**	0.42 (0.10; 1.71)	**0.21** **(0.07; 0.66)**	0.52 (0.16; 1.66)
Prednisolone	1.94 (0.90; 4.19)	2.21 (0.99; 4.94)	**1.54 ** **(1.02; 2.32)**	1.19 (0.79; 1.81)	**1.62** **(1.12; 2.32)**	1.30 (0.90; 1.87)

* Unadjusted and age-adjusted Cox regression analyses

At the date of the first available questionnaire. Bold text indicates statistically significant results

n: number of patients with at least one answered questionnaire after exclusion of patients with fractures before baseline; HR: Hazard Ratio; CI: Confidence Interval; RA: Rheumatoid Arthritis; RF: Rheumatoid Factor; HAQ: Health Assessment Questionnaire; VAS: Visual Analogue Scale; bDMARDs: biologic Disease-modifying Antirheumatic Drugs; SD: Standard Deviation; NA: Not Applicable; NA^1^: Not Applicable since proportional hazards assumptions were not fulfilled

Since there was no excess risk of fractures in newly diagnosed RA patients compared with controls, no predictor analyses were performed for this group. In patients with RA since five or more years at study start, the results of predictor analyses were largely similar to the results of the total cohort (Supplementary Tables 7 and 8, Additional file 1). In this group, there were too few fractures in patients with bDMARDs for meaningful analyses.

## Discussion

The estimated incidence of fractures in this community-based cohort of patients with RA was just over 1 per 100 person-years, which is within the range of what earlier RA studies have found [5]. As expected women had higher incidences of fractures in general but both men and women with RA had increased risk of fractures compared with the general population. The association between hip fractures and RA was strongest in men, especially in analyses of patients with established RA only. This pattern was also indicated in a Spanish study from 2018, where the female to male ratio of hip fractures in RA patients was almost 1:1 at the end of the study period, in contrast to the general population where the ratio was about 3.6:1. The authors of this study also noted that men with RA suffered from their first hip fracture at the same age as women in contrast to men without RA who had fractures on average 5–10 years later than the corresponding women [9]. A somewhat stronger association between hip fractures and RA in men was also seen in a large cohort study based on administrative claims data from the United States [22].

There have been a number of attempts to assess differences in disease characteristics, treatment choices and treatment outcomes in men and women with RA, but the interpretation of the results is complex. Consistent with the literature [23–26], women with RA in this study scored somewhat higher in HAQ and VAS for pain and global health, but the differences in other baseline characteristics were not striking. More women than men both with and without RA had had fractures before study start and were excluded from the statistical analyses. Since this could have led to underestimation of the long term fracture risk in women with RA, sensitivity analyses where patients with previous osteoporosis-related fractures were not excluded, but instead such fractures were adjusted for, were performed, but the results did not change substantially, neither in men nor in women.

Studies of bone mass in RA patients have shown accelerated bone loss not only in women but also in men with RA [11, 13], starting already early in disease [12]. This could be an explanation for the higher risk of fractures in RA patients of both sexes. Men are generally examined and treated for osteoporosis to a lesser extent than women [15], and this is also true for men with RA [27]. In the light of the doubled mortality rates after hip fractures in men compared with women [9, 15], osteoporosis in men (with and without RA) is in need of more attention. Falls are important risk factors of fractures and RA patients have been reported to have high risk of falling [4, 28, 29]. In contrast to the general population, studies of patients with RA have not reported any major influence of age or sex on the risk of falls [29], indicating that men with RA may fall as much as women with RA do. This could be part of the explanation for the limited difference in the rate of hip fractures between men and women with RA.

The risk of fractures early in disease has not been extensively evaluated before, although there are two studies finding a high risk of osteoporosis-related fractures the first years after disease onset [8, 14]. In this study we had the opportunity to follow 738 patients from their first year of diagnosis to a maximum of 10 years later and compare the risk of fractures to matched general population controls. In this subcohort, there was no statistically significant excess risk of fractures, except for fractures in the proximal upper arm. On the other hand, risk estimates were higher in the substudy of patients with more than 5 years disease duration of RA in 1997, suggesting that increased risk of fractures is in particular seen in patients with established disease. As a statistical measure of this, there was a trend towards an interaction between RA and early/established subcohort status, in particular for hip fractures, although it did not reach statistical significance. Patients with early RA (and their controls) were younger and had substantially less fractures than the patients with established disease. This could result in low power in the statistical analyses on early RA. Nevertheless, the results on early RA patients could also be partly due to the different inclusion period (1997 to 2006) compared to patients with established RA already at study start. The early RA patients had been differently treated both for their RA (with earlier and more extensive use of methotrexate and bDMARDs) and potentially also for the risk of osteoporosis. Although there most likely were many different changes in clinical practice over time during the study period, the organization of the tax funded health care system in the area was essentially unchanged, with a single hospital providing most secondary out-patient and all in-patient care. However, it should be noted that the present findings of no increased risk of fractures in patients diagnosed with RA after 1996 contrast with the lack of obvious reduction of the incidence of fractures over time in RA patients in earlier studies [5, 8].

In an attempt to come closer to the definition of fragility fractures, supplementary analyses, in which fractures with external cause ICD-10 codes for high-energy trauma during the study period were excluded, were performed. However, the traumatic fractures were few and the results turned out similar, except for the results in men, which were no longer significant. In men with RA there were just 51 fractures in total during the study period and likely, when 5 of these were classified as traumatic, the analyses of this subset became underpowered. In men with established disease there was again a strong association between RA and fractures, especially in the hip.

When examining potential baseline predictors of fractures in the RA patients, as expected age was the most important predictor of fractures. In accordance with the results of enhanced associations between established RA and fractures, longer RA duration was associated with higher risks of fractures, as well as higher scores for HAQ and VAS global health. These results are in line with observations in earlier studies [5, 14] although disease duration and HAQ scores may be partly influenced by age which has also been the case in earlier studies [30]. Treatment with glucocorticosteroids at baseline on the other hand did not show a statistically significant association with fractures after adjustment for age. There are ongoing discussions of the benefits and harms of glucocorticosteroids on bone health, and a common opinion is that it is a question of dose, length of treatment and underlying indication [2]. In this study information on daily doses of Prednisolone was unavailable (although common practice at the time was to use doses of 5 to 7.5 mg, as shown in a report on an early RA inception cohort [13]), which is a major limitation. Dose variation between patients and over time could be a reason for lack of association with fractures. Additional limitations apply. First, baseline data in this cohort was limited. Other variables that would have been interesting to include, but were unavailable, were for instance anti-citrullinated peptide antibody (ACPA) status and composite disease activity scores, as well as several established risk factors for fractures in the general population (e.g. smoking, alcohol use, body mass index, menopause and comorbidities), prevalent osteoporosis diagnosis and anti-osteoporotic treatment. Also, information on socioeconomic status and ethnicity would have been valuable since these factors covary with both RA severity and the risk of osteoporosis-related fractures [31, 32]. Although the study was performed in a single city, some variability in ethnicity and socioeconomic status was likely present in this study, and may have affected the results. Second, the method of detecting fractures, from registry databases on inpatient care, with no verification with radiographic documentation, constitutes a limitation. Radiographic examination is especially important for detecting vertebral fractures, but we cannot for certain rule out misclassification of other fractures as well. The Swedish National Patient Register has been validated several times, both through reviews of patient records and by comparison with the Swedish quality register for hip fractures (the Swedish Hip Fracture Register), showing consistently good results regarding the validity of hip fracture diagnoses [33, 34]. However, other fracture types may be managed in outpatient care to a greater extent, and not captured in this study. Third, the number of men with early RA was limited and the number of fractures was insufficient for further analyses of fracture risk in this group of patients. Further studies on this specific patient group would be valuable since information is lacking and there is a risk that these patients are missed in fracture prevention work.

A strength of this study is the use of a community-based cohort with all known RA patients in the area, including all types of patients seen in clinical practice. The patients were treated according to the general recommendations during the studied time period, and the cohort should be representative for most other patients with similar health care opportunities and living conditions during the study period. The subanalyses of the patients with short disease duration gave us the opportunity to look into the risk of fractures in early RA and compare the results to those of more established RA patients.

## Conclusions

Both men and women with RA had increased risk of osteoporosis-related fractures compared with the general population. Men with established disease had particularly high risk of hip fractures, and more focus on fracture prevention in this patient group would likely be beneficial. Patients with new onset of disease between 1997 and 2006 were not at significantly increased risk of fractures overall or hip fractures during the first ten years after diagnosis, but no conclusions on fracture risk in men with early RA could be drawn, and this should be further studied.

### Electronic supplementary material

Below is the link to the electronic supplementary material.

Supplementary Material 1

## Data Availability

The datasets used and/or analysed during the current study are available from the corresponding author on reasonable request.

## Abbreviations

RA: Rheumatoid arthritis
CI: Confidence intervals
HR: Hazard ratio
HAQ: Health assessment questionnaire
BMI: Body mass index
VAS: Visual analogue scale
bDMARDs: Biologic disease-modifying antirheumatic drugs
ICD: International Classification of Diseases
RF: Rheumatoid factor
PYR: Person years of risk
