# Supplementary material for: Osteoporosis-related fractures in men and women with established and early rheumatoid arthritis: predictors and risk compared with the general population
Source: BMC Rheumatol. 2023 Sep 8;7:28. doi: 10.1186/s41927-023-00354-7 (PMC10486097; doi:10.1186/s41927-023-00354-7)
Supplement: Supplementary file 1 — Supplementary Material 1 [file 41927_2023_354_MOESM1_ESM.docx]

Supplementary table 1. ICD codes for osteoporosis-related fractures used in this study.

| **Fracture site** | **ICD 9** | **ICD 10** |
| --- | --- | --- |
| Hip | 820 | S720, S721, S722 |
| Vertebral column | 805C, 805D, 805E, 805F | S220, S221, S320, S327 |
| Proximal upper arm | 812A, 812B | S422 |
| Distal forearm | 813E, 813F | S525, S526, S528 |

Supplementary table 2. Baseline characteristics of patients with osteoporosis-related fractures before study start.

| **Patients with previous fractures** | **Men** | **Women** | **All** |
| --- | --- | --- | --- |
| Number | 13 | 81 | 94 |
| Age (years) mean (SD) | 76.9 (8.3) | 75.2 (7.7) | 75.4 (7.7) |
| RA duration at inclusion (years), median (IQR) | 14 (5-22) | 14 (5-28) | 14 (5-27) |
| RA duration at first questionnaire (years), median (IQR) | 17 (7-27) | 15 (7-28) | 17 (7-28) |
| RF-positive n (%) | 9 (81.8) | 52 (82.5) | 61 (82.4) |
| HAQ mean (SD)* | 1.44 (0.92) | 1.72 (0.89) | 1.69 (0.90) |
| VAS pain (mm) mean (SD)* | 39.5 (25.6) | 53.0 (29.5) | 51.3 (29.2) |
| VAS global health (mm) mean (SD)* | 41.0 (30.5) | 49.1 (29.6) | 48.1 (29.5) |
| Methotrexate n (%)* | 2 (25.0) | 20 (32.3) | 22 (31.4) |
| csDMARDs other than Methotrexate n (%)* | 2 (25.0) | 12 (19.4) | 14 (20.0) |
| bDMARDs n (%)* | 0 (0.0) | 1 (1.6) | 1 (1.4) |
| Prednisolone n (%)* | 4 (50.0) | 14 (22.6) | 18 (25.7) |

* At the date of the first available questionnaire.
SD: Standard Deviation; IQR: Interquartile Range; RA: Rheumatoid Arthritis; RF: Rheumatoid Factor; HAQ: Health Assessment Questionnaire; VAS: Visual Analogue Scale; csDMARDs: conventional synthetic Disease-modifying Antirheumatic Drugs; bDMARDs: biologic Disease-modifying Antirheumatic Drugs.

Supplementary table 3. Baseline characteristics in patients not returning any baseline questionnaire during the study period.

|  | Men (n 117) | Women (n 288) | All (n 405) |
| --- | --- | --- | --- |
| Age (years) mean (SD) | 61.2 (17.9) | 63.5 (16.5) | 62.8 (16.9) |
| RA duration at inclusion (years), median (IQR) | 0 (0; 13) | 3 (0; 14) | 2 (0; 14) |
| RF-positive n (%) | 60 (65.9) | 179 (72.2) | 239 (70.5)* |
| bDMARDs n (%) | 0 (0.0) | 1 (0.3) | 1 (0.2) |
| Previous osteoporosis-related fracture n (%) | 5 (4.3) | 19 (6.6) | 24 (5.9) |

n: Number; SD: Standard Deviation; RA: Rheumatoid Arthritis; IQR: Interquartile Range; RF: Rheumatoid Factor; bDMARDs: biologic Disease-modifying Antirheumatic Drugs.

*66 patients had missing information on RF

Supplementary table 4. Number and incidence of vertebral, proximal upper arm and distal forearm fractures in patients and controls.

| Fracture |  | Patients | | | Controls | | |
| --- | --- | --- | --- | --- | --- | --- | --- |
| **Total cohort** |  | **Men** | **Women** | **All** | **Men** | **Women** | **All** |
| Vertebral column | n (%) | 5 (0.98) | 16 (1.19) | 21 (1.13) | 12 (0.66) | 50 (0.99) | 62 (0.91) |
|  | Incidence/1000 PY (95% CI) | 0.75  (0.24; 1.74) | 0.85  (0.49; 1.38) | 0.82  (0.51; 1.26) | 0.47  (0.24; 0.82) | 0.65  (0.48; 0.86) | 0.60  (0.46; 0.77) |
| Proximal upper arm | n (%) | 5 (0.98) | 46 (3.42) | 51 (2.75) | 11 (0.61) | 107 (2.13) | 118 (1.72) |
|  | Incidence/1000 PY (95% CI) | 0.75  (0.24; 1.75) | 2.46  (1.80; 3.28) | 2.01  (1.50; 2.64) | 0.43  (0.21; 0.77) | 1.40  (1.15; 1.69) | 1.16  (0.96; 1.38) |
| Distal forearm | n (%) | 4 (0.78) | 27 (1.99) | 31 (1.66) | 14 (0.77) | 105 (2.10) | 119 (1.75) |
|  | Incidence/1000 PY (95% CI) | 0.60  (0.16; 1.53) | 1.43  (0.94; 2.08) | 1.21  (0.82; 1.72) | 0.55  (0.30; 0.92) | 1.38  (1.13; 1.67) | 1.17  (0.97; 1.40) |
| **Early RA*** |  |  |  |  |  |  |  |
| Vertebral column | n (%) | 0 (0.00) | 5 (0.98) | 5 (0.70) | 3 (0.37) | 8 (0.39) | 11 (0.39) |
|  | Incidence/1000 PY (95% CI) | 0  (-) | 1.06  (0.34; 2.48) | 0.77  (0.25; 1.80) | 0.43  (0.09; 1.27) | 0.43  (0.19; 0.85) | 0.43  (0.22; 0.77) |
| Proximal upper arm | n (%) | 1 (0.49) | 10 (1.96) | 11 (1.54) | 5 (0.62) | 17 (0.84) | 22 (0.78) |
|  | Incidence/1000 PY (95% CI) | 0.57  (0.01; 3.15) | 2.13  (1.02; 3.92) | 1.70  (0.85; 3.05) | 0.72  (0.23; 1.69) | 0.92  (0.54; 1.47) | 0.87  (0.54; 1.31) |
| Distal forearm | n (%) | 0 (0.00) | 5 (0.98) | 5 (0.70) | 6 (0.74) | 22 (1.09) | 28 (0.99) |
|  | Incidence/1000 PY (95% CI) | 0  (-) | 1.06  (0.35; 2.48) | 0.77  (0.25; 1.80) | 0.87  (0.32; 1.89) | 1.20  (0.75; 1.82) | 1.11  (0.74; 1.60) |
| **Established RA**** |  |  |  |  |  |  |  |
| Vertebral column | n (%) | 4 (1.77) | 8 (1.32) | 12 (1.44) | 5 (0.69) | 28 (1.31) | 33 (1.15) |
|  | Incidence/1000 PY (95% CI) | 1.42  (0.39; 3.63) | 0.99  (0.43; 1.95) | 1.10  (0.57; 1.92) | 0.46  (0.15; 1.08) | 0.82  (0.55; 1.19) | 0.73  (0.51; 1.03) |
| Proximal upper arm | n (%) | 1 (0.44) | 24 (3.96) | 25 (2.99) | 3 (0.41) | 59 (2.76) | 62 (2.17) |
|  | Incidence/1000 PY (95% CI) | 0.35  (0.01; 1.96) | 3.01  (1.93; 4.48) | 2.31  (1.50; 3.41) | 0.28  (0.06; 0.81) | 1.74  (1.32; 2.24) | 1.39  (1.06; 1.78) |
| Distal forearm | n (%) | 3 (1.31) | 10 (1.61) | 13 (1.53) | 2 (0.28) | 54 (2.54) | 56 (1.96) |
|  | Incidence/1000 PY (95% CI) | 1.06  (0.22; 3.11) | 1.23  (0.59; 2.26) | 1.19  (0.63; 2.03) | 0.19  (0.02; 0.67) | 1.60  (1.20; 2.09) | 1.26  (0.95; 1.63) |

RA: Rheumatoid Arthritis; n: Number; PY: Person Years; CI: Confidence Interval.

*Early RA: newly diagnosed (within 1 year) patients from the year of 1997 with follow-up time maximal 10 years.

**Established RA: patients with RA diagnosis for ≥5 years at study start (1997).

Supplementary table 5. IRR and HR for vertebral, proximal upper arm and distal forearm fractures in RA patients compared with matched controls.

|  | INCIDENCE RATE RATIO (95% CI) | | | HAZARD RATIO (95% CI) | | |
| --- | --- | --- | --- | --- | --- | --- |
| **Total cohort** | **Men** | **Women** | **All** | **Men** | **Women** | **All** |
| Vertebral column | 1.60 (0.44; 5.10) | 1.31 (0.69; 2.32) | 1.36 (0.79; 2.25) | NA^2^ | 1.55  (0.85; 2.83) | 1.48  (0.86; 2.53) |
| Proximal upper arm | 1.74 (0.74; 5.75) | **1.76 (1.22; 2.50)** | **1.74 (1.23; 2.42)** | NA^2^ | **2.11  (1.43; 3.12)** | **2.12  (1.46; 3.08)** |
| Distal forearm | NA^1^ | 1.04 (0.65; 1.59) | 1.04 (0.67; 1.54) | NA^2^ | 1.10 0.69; 1.75) | 1.05  (0.68; 1.62) |
| **Early RA*** |  |  |  |  |  |  |
| Vertebral column | NA^1^ | 2.46  (0.63; 9.60) | 1.79 (0.49; 5.90) | NA^2^ | NA^2^ | NA^2^ |
| Proximal upper arm | NA^1^ | 2.32  (0.95; 5.39) | 1.97 (0.86; 4.22) | NA^2^ | **2.47 (1.09; 5.57)** | **2.35 (1.09; 5.08)** |
| Distal forearm | NA^1^ | 0.89  (0.26; 2.43) | 0.70  (0.21; 1.84) | NA^2^ | NA^2^ | NA^2^ |
| **Established RA**** |  |  |  |  |  |  |
| Vertebral column | NA^1^ | 1.21 (0.48; 2.72) | 1.50 (0.70; 2.96) | NA^2^ | NA^2^ | 1.76 (0.84; 3.70) |
| Proximal upper arm | NA^1^ | **1.73**  **(1.03; 2.80)** | **1.67**  **(1.00; 2.67)** | NA^2^ | **2.26 (1.28; 3.99)** | **2.32 (1.32; 4.07)** |
| Distal forearm | NA^1^ | 0.77  (0.35; 1.52) | 0.94 (0.47; 1.74) | NA^2^ | 0.81 (0.39; 1.68) | 0.93 (0.48; 1.81) |

Bold text indicates statistically significant results. IRR: Incidence Rate Ratio; HR: Hazard Ration; RA: Rheumatoid Arthritis; CI: Confidence Interval; NA^1^: Not applicable due to <5 events in patients with RA in the IRR analysis. NA^2^: Not applicable due to <10 events in patients with RA in the Cox regression analysis.

*Early RA: newly diagnosed (within 1 year) patients from the year of 1997 with follow-up time maximal 10 years.

**Established RA: patients with RA diagnosis for ≥5 years at study start (1997).

Supplementary table 6. Hazard ratios for osteoporosis-related fractures in RA patients compared with matched controls, fractures with external cause ICD-10 codes for high-energy trauma during the study period excluded.

|  | HAZARD RATIO (95% CI) | | |
| --- | --- | --- | --- |
| **Total cohort** | **Men** | **Women** | **All** |
| Hip fracture | 1.49 (0.90; 2.46) | **1.38 (1.11; 1.72)** | **1.40 (1.15; 1.71)** |
| Fractures in total | 1.41 (0.92; 2.16) | **1.53 (1.27; 1.84)** | **1.51 (1.27; 1.79)** |
| **Early RA*** |  |  |  |
| Hip fracture | NA | 0.76 (0.42; 1.38) | 0.72 (0.42; 1.21) |
| Fractures in total | NA | 1.10 (0.71; 1.72) | 0.97 (0.65; 1.45) |
| **Established RA**** |  |  |  |
| Hip fracture | **3.20 (1.48; 6.92)** | **1.73 (1.28; 2.35)** | **1.88 (1.42; 2.49)** |
| Fractures in total | **2.60 (1.34; 5.05)** | **1.81 (1.38; 2.37)** | **1.90 (1.48; 2.44)** |

Bold text indicates statistically significant results. RA: Rheumatoid Arthritis; CI: Confidence Interval; NA: Not applicable due to <10 events in patients with RA.

*Early RA: newly diagnosed (within 1 year) patients from the year of 1997 with follow-up time maximal 10 years.

**Established RA: patients with RA diagnosis for ≥5 years at study start (1997).

Supplementary table 7. Baseline predictors of osteoporosis-related fractures overall in established RA patients. Unadjusted and age-adjusted Cox regression analyses.

| **Fractures overall** | **Men** | | **Women** | | **All** | |
| --- | --- | --- | --- | --- | --- | --- |
|  | HR (95% CI) | Age-adjusted  HR (95% CI) | HR (95% CI) | Age-adjusted  HR (95% CI) | HR (95% CI) | Age-adjusted  HR (95% CI) |
| Age, per 10 years | **5.78  (3.34; 10.01)** | NA | **2.23 (1.79; 2.76)** | NA | **2.64  (2.15; 3.23)** | NA |
| RA duration, per 10 years | 1.28  (0.94; 1.75) | 0.95 (0.71; 1.27) | **1.20  (1.02; 1.42)** | 1.00 (0.85; 1.18) | **1.22  (1.05; 1.41)** | 0.99  (0.86; 1.14) |
| RF-positive | 1.27  (0.44; 3.69) | 1.52  (0.52; 4.46) | NA^1^ | NA^1^ | NA^1^ | NA^1^ |
| HAQ, per SD | 1.20  (0.81; 1.78) | 1.03  (0.70; 1.53) | **1.36 (1.07; 1.73)** | 1.12  (0.88; 1.43) | **1.31  (1.07; 1.60)** | 1.08 (0.88; 1.32) |
| VAS pain, per SD | 1.04  (0.72; 1.50) | 1.41 (0.96; 2.06) | 1.01  (0.81; 1.27) | 0.91  (0.72; 1.15) | 1.02  (0.84; 1.24) | 0.98  (0.81; 1.20) |
| VAS global health, per SD | 1.17  (0.81; 1.69) | 1.46 (0.98; 2.18) | 1.09 (0.87; 1.38) | 0.98 (0.77; 1.25) | 1.12 (0.92; 1.36) | 1.05 (0.86; 1.28) |
| Methotrexate | 0.51  (0.19; 1.34) | 0.64 (0.24; 1.70) | 0.89  (0.55; 1.44) | 1.02  (0.63; 1.66) | 0.78  (0.51; 1.20) | 0.91  (0.59; 1.40) |
| bDMARDs | NA^2^ | NA^2^ | NA^2^ | NA^2^ | NA^2^ | NA^2^ |
| Prednisolone | 2.05  (0.95; 4.43) | **2.36  (1.05; 5.29)** | 1.51 (0.92; 2.47) | 1.15  (0.70; 1.90) | **1.62  (1.08; 2.45)** | 1.31  (0.87; 1.99) |

At the date of the first available questionnaire. Bold text indicates statistically significant results.
HR: Hazard Ratio; CI: Confidence Interval; RA: Rheumatoid Arthritis; RF: Rheumatoid Factor; HAQ: Health Assessment Questionnaire; VAS: Visual Analogue Scale; bDMARDs: biologic Disease-modifying Antirheumatic Drugs; SD: Standard Deviation; NA: Not Applicable. NA^1^: Not Applicable since proportional hazards assumptions were not fulfilled; NA^2^: Not applicable due to less than 5 events in patients with established RA on bDMARDs.

Supplementary table 8. Baseline predictors of hip fractures in established RA patients. Unadjusted and age-adjusted Cox regression analyses.

| **Hip fractures** | **Men (n 183)** | | **Women (n 463)** | | **All (n 646)** | |
| --- | --- | --- | --- | --- | --- | --- |
|  | HR (95% CI) | Age-adjusted  HR (95% CI) | HR (95% CI) | Age-adjusted  HR (95% CI) | HR (95% CI) | Age-adjusted  HR (95% CI) |
| Age, per 10 years | **5.51  (3.17; 9.56)** | NA | **2.27  (1.76; 2.91)** | NA | **2.74 (2.18; 3.46)** | NA |
| RA duration, per 10 years | 1.30  (0.93; 1.81) | 0.88  (0.64; 1.21) | 1.10  (0.90; 1.34) | 0.93  (0.76; 1.13) | 1.14  (0.96; 1.35) | 0.93 (0.79; 1.09) |
| RF-positive | 2.38  (0.56; 10.2) | **3.36  (0.77; 14.69)** | NA^1^ | NA^1^ | **2.34  (1.07; 5.11)** | **2.79  (1.28; 6.11)** |
| HAQ, per SD | 1.19  (0.78; 1.83) | 1.01  (0.66; 1.56) | 1.23  (0.94; 1.60) | 1.00  (0.76; 1.31) | 1.20  (0.96; 1.50) | 0.98  (0.78; 1.23) |
| VAS pain, per SD | 1.02  (0.68; 1.52) | 1.27  (0.84; 1.91) | 1.04  (0.80; 1.34) | 0.93 (0.71; 1.21) | 1.03 (0.83; 1.27) | 0.98 (0.78; 1.21) |
| VAS global health, per SD | 1.22  (0.82; 1.81) | 1.34  (0.90; 2.01) | 1.05 (0.80; 1.36) | 0.93  (0.71; 1.22) | 1.09 (0.88; 1.36) | 1.01  (0.80; 1.26) |
| Methotrexate | 0.46  (0.16; 1.37) | 0.66 (0.22; 1.97) | 0.92  (0.53; 1.59) | 1.04 (0.60; 1.80) | 0.78  (0.48; 1.26) | 0.90 (0.56; 1.47) |
| bDMARDs | NA^2^ | NA^2^ | NA^2^ | NA^2^ | NA^2^ | NA^2^ |
| Prednisolone | 1.91  (0.82; 4.42) | 2.03  (0.84; 4.91) | 1.54  (0.88; 2.70) | 1.19  (0.67; 2.09) | **1.64  (1.03; 2.60)** | 1.31  (0.82; 2.09) |

At the date of the first available questionnaire. Bold text indicates statistically significant results.
HR: Hazard Ratio; CI: Confidence Interval; RA: Rheumatoid Arthritis; RF: Rheumatoid Factor; HAQ: Health Assessment Questionnaire; VAS: Visual Analogue Scale; bDMARDs: biologic Disease-modifying Antirheumatic Drugs; SD: Standard Deviation; NA: Not Applicable; NA^1^: Not Applicable since proportional hazards assumptions were not fulfilled; NA^2^: Not applicable due to less than 5 events in patients with established RA on bDMARDs.
